# Expanding protection motivation theory: investigating an application to animal owners and emergency responders in bushfire emergencies

**DOI:** 10.1186/s40359-017-0182-3

**Published:** 2017-04-26

**Authors:** Rachel Westcott, Kevin Ronan, Hilary Bambrick, Melanie Taylor

**Affiliations:** 1 Centre for Health Research, School of Medicine, Western Sydney University, Campbelltown Sydney, Australia; 2 Bushfire and Natural Hazards Cooperative Research Centre, Melbourne, Australia; 3 Clinical Psychology, School of Health, Medical and Applied Sciences Central Queensland University, Rockhampton, QLD Australia; 4 School of Public Health and Social Work, Queensland University of Technology, Victoria Park Road, Kelvin Grove, QLD Australia; 5 Department of Psychology, Macquarie University, Sydney, Australia

**Keywords:** Protection motivation theory, Animals, Animal owners, Emergency responders, Bushfire, Wildfire, Natural hazards, Preparedness

## Abstract

**Background:**

Protection Motivation Theory (PMT) was developed by Rogers in 1975, to describe how individuals are motivated to react in a self-protective way towards a perceived health threat. Rogers expected the use of PMT to diversify over time, which has proved true over four decades. The purpose of this paper is to explore how PMT can be used and expanded to inform and improve public safety strategies in natural hazards. As global climate change impacts on the Australian environment, natural hazards seem to be increasing in scale and frequency, and Emergency Services’ public education campaigns have necessarily escalated to keep pace with perceived public threat. Of concern, is that the awareness-preparedness gap in residents’ survival plans is narrowing disproportionately slowly compared to the magnitude of resources applied to rectify this trend. Practical applications of adaptable social theory could be used to help resolve this dilemma.

**Discussion:**

PMT has been used to describe human behaviour in individuals, families, and the parent-child unit. It has been applied to floods in Europe and wildfire and earthquake in the United States. This paper seeks to determine if an application of PMT can be useful for achieving other-directed human protection across a novel demographic spectrum in natural hazards, specifically, animal owners and emergency responders in bushfire emergencies.

These groups could benefit from such an approach: owners to build and fortify their response- and self-efficacy, and to help translate knowledge into safer behaviour, and responders to gain a better understanding of a diverse demographic with animal ownership as its common denominator, and with whom they will be likely to engage in contemporary natural hazard management. Mutual collaboration between these groups could lead to a synergy of reciprocated response efficacy, and safer, less traumatic outcomes.

**Summary:**

Emergency services’ community education programs have made significant progress over the last decade, but public safety remains suboptimal while the magnitude of the awareness-preparedness gap persists. This paper examines an expanded, other-directed application of PMT to expand and enhance safer mitigation and response behaviour strategies for communities threatened by bushfire, which may ultimately help save human life.

**Table:** 

*If you are an able bodied person on your own with one cat then it’s simple – have a backpack ready, put the cat in a carrier and you’re away in about 30 seconds. If you’re a single mum with an autistic child and an assistance dog, and you have Nanna on Tuesdays and you have six chooks, two ponies, three dogs and goldfish, you’re better off starting in about September*. South Australian Country Fire Service Community Engagement Officer Therese Pedler, 2015.	

## Background

Bushfires are increasing in Australia and worsening globally within temporal and geographic parameters: the corollary of climate change and increasing severe weather events [1, 2]. Fire can become an emergency when people, property, the environment and other assets are affected: the animal-owning public faces this challenge alongside the need to properly and safely manage their animals in addition to themselves. Animal owners are a diverse and widespread group whose needs have not been specifically examined in the context of bushfire, despite the growing understanding of the strong link between effective animal management in an emergency and the saving of human life [3, 4]. In addition, while approximately 63% of Australian households own companion animals [5], the number of animals owned by primary producers, and in animal oriented businesses, is much larger [6].

Prevention and preparedness, the prerequisites for effectively managing a (bushfire) risk, are widely documented as being poorly implemented across all hazards [7–10] whether animals are involved or not. Although community awareness of the danger posed by bushfires seems to be increasing [11], messages of hazard mitigation and preparedness still are inconsistently received, despite the escalation of Emergency Services’ public education campaigns throughout the last decade. The *awareness-preparedness gap* in community and individual residents’ survival plans is narrowing disproportionately slowly compared to the magnitude of resources utilised to reverse this trend. Thus, new strategies and tactics which resonate broadly with people in at-risk areas and demographics need to be identified and implemented, to accelerate the transition from awareness into action [9, 12]. The impetus is human health and safety, and in simple economic terms, prevention and preparedness are vastly less costly than response and recovery [13, 14].

The presence of animals adds varying degrees of complexity to owners’ preparedness and planning when faced with an emergency such as fire or flood. Any subsequent reluctance or delay in adopting safe and timely behaviour can lead to injury or even loss of life, and further, risk the lives of emergency responders. Synergistic collaboration promoting shared responsibility, self-sufficiency and a deeper reciprocal understanding between emergency responders and animal owners can build trust, promote community engagement and strengthen a community’s capacity to respond and recover [12, 15].

To date, the majority of the academic literature about animal owners in disasters is skewed towards the retrospective experiences only of pet owners [16]. This omits to document the interaction between animal owners and emergency responders during an incident involving many species of animals owned in a variety of contexts, or present in wildlife habitat. Consequently, there is a need to investigate these different groups to fill current gaps in emergency communication and warnings, either within, or beyond, both groups [17, 18]. This paper contributes to filling this gap. It explores an application of Protection Motivation Theory (PMT) to better theorise and understand the behaviour of animal owners in bushfire to facilitate targeted and meaningful preparedness initiatives and motivate the translation of knowledge into effective, adaptive action.

A case study of a bushfire at-risk regional centre located on the Lower Eyre Peninsula in South Australia – commonly referred to as “the driest state in the driest continent” [19] will be used to investigate the interactions and challenges facing animal owners and emergency responders, and to determine if an application and expansion of PMT can contribute to new or enhanced mitigation and preparedness measures which can be integrated into current arrangements to promote human safety and support community well-being.

Several distinguishing factors determined the research site. These include (i) the area’s recent and severe fire history; (ii) regional people tend to appear more resourceful and self-reliant than their urban counterparts [4, 20–23]; (iii) the diversity of animal owners; and (iv) geographical location - it is distant enough from large cities to require some effort and expense to visit, and hence is not “over” researched.

Animal owners overall are a diverse group who could include owners of one or several animals, primary producers, animal Small to Medium Enterprise (SME) operators and guardians or custodians of wildlife. In addition to firefighters, emergency responders can include police, rescue officers of the State Emergency Service (SES), staff of the Royal Society for the Prevention of Cruelty to Animals (RSPCA), Government agriculture officers, Department of Environment rangers, veterinarians and other stakeholders. The ‘unpublished observations’ noted in this paper are not part of data collection. These are essentially personal communications from some stakeholders during preliminary investigations for research design.

A pragmatic approach within a critical realist ontology and contextualist, experiential epistemology guided this qualitative research design, due to the need to arrive at practical answers to issues of policy and practice [24–26]. Thematic Analysis (TA) [25, 27] was chosen for data analysis because it is a flexible, versatile method which is independent of theory (7, 20). This allows for extraction of detailed, experiential material from the data to examine in the context of the application and proposed expansion of PMT.

Using PMT, a robust and flexible social theory, animal owners of all categories may be assisted to better understand their own response behavior ahead of seasonal danger, so that it becomes safer, instinctual and routine. The ability to translate awareness into effective planning and preparedness well before the superimposed pressures of an imminent threat arrive, and to collaboratively engage with emergency responders and the community, may help to significantly narrow the gap between hazard awareness and hazard survival.

In his 1983 revision of PMT, Rogers noted that he expected new and different applications for his theory to be developed in the future [28–30]. This has proved to be true over four decades, evolving into disciplines beyond the health sector.

This paper reviews how the use of PMT has evolved beyond self-directed health applications, and explores its potential relevance to animal owners in the context of bushfire. Consequently, it proposes an extension to the theory with respect to other-directed human behavior in natural hazard emergencies. The corollary of this extension aims to be practical, and testable, applications by emergency responders to assist in community engagement, and to improve natural hazard preparedness and planning for animal owners and/or in the presence of animals. A strength, and test, of adaptable, versatile social theory is its ability to successfully “bridge exploration and problem-solving” (Akama, Y. personal communication 2015). Actively applying theory to enquiry, and using the results to form practical strategies beneficial to animal owners *and others*, could help narrow the awareness-preparedness gap overall, and illuminate other research possibilities.

### Protection Motivation theory – genesis and early development

Protection Motivation Theory, [28] was originally developed for the health promotion and disease prevention sector, and describes how individuals are motivated to react in a protective way towards a perceived threat. It has four key elements: “threat appraisal”, followed by “coping appraisal”, which comprises “response efficacy” – the belief that certain processes *will mitigate* the threat - and “self-efficacy”, an individual’s idea of their *own ability* to implement the required actions to mitigate the threat.

Rogers listed four key elements of PMT thus:
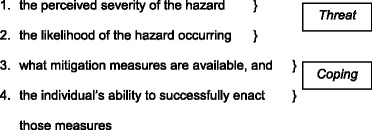


Protection Motivation Theory can be applied to “any threat for which there is an effective recommended response that can be carried out by the individual” [31]. Maddux and Rogers [29] found self-efficacy to be “the most powerful predictor of behavioural intentions” that precede actual behavior [10]. A robust self-efficacy is more likely to (i) lead to the taking of protective action in an appropriate timeframe, (ii) influence the degree of receptivity to information and (iii) promote the likelihood of taking effective remedial action [12, 32].

The objective of PMT is to recognise and assess the danger, and then counter this assessment with effective and efficacious mitigation options. This makes PMT applicable to many social problems; it has been applied to studies of natural hazards - earthquake in the United States, and flood in Germany and France [33–35], as well as adaptations to climate change [36, 37]. This is consistent with Rogers’ observation in his 1983 revision that other factors could influence protection motivation and coping behaviors of individuals and groups.

Protection Motivation Theory is recognised as a more mature, sophisticated and humane process than its sometimes controversial [38] predecessor, *Fear Appeals* theory. Tanner [39] explains how frightening the target audience “is not the objective – promoting responsible behaviours is”. Using fear as a motivator eventually plateaus and becomes ineffective, and fails to advocate for positive outcome expectancy, or inform how this might be achieved.

Rogers’ 1983 revision of PMT [30] produced a more comprehensive model which included adaptive response costs and maladaptive response rewards in the cognitive mediation equation. This resonates significantly among animal owners facing complex decisions in the variably complex environment of their own social microclimate. Precisely because of this relevance to animal owners as a demographic sharing a core commonality which is anecdotally repeatedly reported as being problematic in emergencies, PMT is a logical avenue to explore in developing an enhanced and expanded emergency response theory. A background review of the animals in emergencies literature follows, preceding a description of PMT, and its advantages in this area.

## Literature review

### The case for considering animals in emergency management

It is widely agreed that animals add enrichment and complexity to modern life [40–44]. In emergencies, the presence of animals may distract, deter or encourage timely and safe behaviour. Recently there has been a resurgence of academic interest in animal emergency management, following a flurry of publications post Hurricane Katrina, the storm system which struck the Louisiana, USA, coast in 2005. The post-Katrina interest waned, but the grey literature remained engaged, as jurisdictions, particularly in Western society, began to understand more about the importance of including animals in emergency planning. This is evidenced in new and amended legislation, Government documents, official reports, documentary accounts of incidents and the evolution of emergency systems and plans [45–49]. Emergency management has become more sophisticated, and has embraced an increasingly humane and holistic regimen that recognises the importance of psychological health, and that empowered communities may be better able to confront and prevail against adversity [17, 18, 50–54].

Pets are routinely described by their owners as “one of the family” [4, 55–60]. Taylor, Lynch et al [60] found that 86% of Australian pet owners, stated that their pets “made them happy”, and 88% said that their pets were “great companions”. The Council of Australian Governments’ National Strategy for Disaster Resilience [15] has provided overarching guidelines for the direction of Australian emergency management, and has embraced all aspects of this discipline, including provision for animals [61]. Given that 63% of Australian households own a companion animal, and that Australians value their companion and non-companion animals highly, animals need to be included as part of formal emergency management plans. This extends well beyond simplistic “animal welfare” in isolation: while this is important, it is far more significant when the context and extent of human-animal relationships is acknowledged and understood [4, 56, 62–66].

### The costs of prevention versus recovery

Devastating large scale events which attract the world’s attention, such as Hurricane Katrina, have been well documented with respect to the destruction and chaos they bring to people, communities and ecosystems. Natural hazards of varying degrees of severity frequently appear in news bulletins, usually, and understandably, reporting primarily on the human tragedy. In developed countries, the last decade of emergency management has seen changes which privilege environmental concerns in an increasingly holistic approach, and recognise that *prevention* is vastly less costly than *recovery*–in economic, social and environmental terms [13, 14, 41].

### Evacuation and relocation of people and pets

*Action inertia* has been described as a “barrier to safe behaviour” [67]. Evacuation failure due to animal ownership (i.e. animals, directly or indirectly, being the cause of the “inertia”), has been discussed for some time in the disaster literature [4, 50, 59, 68–71]. Timely and well-prepared evacuation or voluntary relocation is often one of the main desirable protective behaviours, and is the focus in this investigation of applying PMT to animal owners and emergency responders in bushfire emergencies.

The strength of human-animal relationships can influence readiness to evacuate [4]. Heath [69] found that evacuation failure in households with pets was greater than in households with children. However, in households where animals were generally managed more responsibly, such as with regular visits to the veterinarian, animals were less likely to adversely influence timely evacuation. Hunt [50] notes that while post-Hurricane Katrina legislation has improved evacuation compliance in the United States, animal owners still name pet ownership as an obstacle to leaving a residence in accordance with emergency evacuation notices.

Providing evacuation facilities for pets, preferably accompanied by their owners, presents considerable logistical and public safety challenges. However, the provision of such a facility could be advantageous for longer term human psychological health, given that pet loss has been found to predict Post Traumatic Stress Disorder (PTSD), acute stress and peri-traumatic dissociation [72].

### The importance of place

Attachment to place is an important consideration to help understand why residents choose to live in areas of higher fire danger, and when managing people displaced from their communities and familiar, secure environments [12, 40, 73, 74]. Eriksen, Gill et al [73] and Paton [12] note the significance of this decision to live in areas of higher fire danger - as people seek refuge from the intensity of urban living, the attraction to a place of peace and beauty is strong. An aesthetically pleasing location, chosen because of its flora *and* fauna, is as much a part of experiencing and achieving good mental health as its destruction by fire is the reverse. New residents may or may not possess sufficient rural living experience or skills to live safely in their new location, may be absentee land owners if they commute to city employment, or may have purchased a property in the middle of winter when bushfires seem a distant and unlikely event. Similarly, special needs categories, such as elderly or disabled animal owners, or a single parent with a disabled child and an assistance dog, may not be as prepared and/or require additional help. For these, as well as for logistical and social reasons, relief centres are usually not far from the emergency location. Again, shared responsibility and cooperative collaboration among animal owners and responders could help reduce the stress of some inevitable and unavoidable temporary separation, while freeing up limited resources to assist those who need the most help.

### Human-animal bond, grief and loss

Attachment theory [75] has expanded over time to include relationships between humans and non-human animals [62]. Animals contribute positively to human life, physically and psychologically. They are noted for the provision of unconditional love and non-judgemental behaviours. Joy, sorrow, love and friendship are all qualities attributed to companion animals. They have a role as diffusers of social awkwardness, or as the means by which new relationships and introductions might form. Some animal owners consider themselves closer to a pet than to family, and rate a pet as being more supportive than humans during times of extreme stress [42, 56, 63]. All groups, including emergency responders, who deal with animals in emergencies or disasters are at risk of psychological trauma, and should have access to mental health services that have an understanding and acknowledgement of the importance and complexities of the human-animal bond [4].

Grief and loss following animal deaths is often not given social legitimacy [42, 58], but should be acknowledged and supported. An absence of the expression of grief can lead to unresolved anger and sadness, and may complicate recovery. Human response to the death of a single animal, possibly the only one an individual has owned, may be very different to the devastation experienced by a farmer facing the loss of an entire herd or flock, but is no less valid [41]. For farmers, the loss is much more complex than only the monetary loss of that year’s wool or meat – frequently many generations of a farming family have added to and established valuable animal genetics which are irreplaceable. Even large scale farmers often know the animals in their breeding herds individually by name. Multigenerational family achievement, reputation and therefore legacy to future generations can be destroyed in a bushfire within hours, with sometimes additional tragic consequences.

The Hurricane Katrina response in August 2005 is infamous for the mass human turmoil and displacement which occurred [16, 18, 76]. In many respects, the Hurricane Katrina emergency illuminated the importance of animals in Western society [76] and was a catalyst for passing of the Pets Evacuation and Transportation Standards (PETS) Act [49] in the wake of public outcry over the impact that event had on animals. Leonard and Scammon [63] explain that the rationale behind the PETS Act was to provide increased safety for humans, encouraging animal owners to evacuate in a timely manner, knowing their animals are not forgotten, with animal welfare as a secondary basis for the legislation.

### Challenges

There are a number of challenges to address among animal-owning groups. These were identified in the literature and during preliminary investigations and research design, and include:

- Maladaptive behavior such as optimistic bias, or deferring a decision to act or evacuate by preferring to “wait and see”. Often this wastes valuable life-saving time [34, 37, 67, 77].
- Belief in myth, folklore or rumour, such as the desirability of releasing animals to “escape”, leaving them to wander at large on public roads – risking high impact collision with emergency vehicles, and the associated trauma, injury, and lost time [51, 78, 79].
- Self-responsibility and self-sufficiency – such as planning and finding safe places to relocate animals, which is the owner’s responsibility [46, 80, 81].
- Information seeking and meaningful advice from accurate and trustworthy sources rather than relying on exaggerated or incorrect messaging [7, 55, 73, 82, 83].
- Failure to have and implement a year-round prevention and preparedness activities routine [7, 9, 11, 12].
- Adaptive response “costs” such as inconvenience, versus maladaptive “rewards”, such as devoting time to a personally preferable activity [10, 30, 84].

These challenges, while not necessarily exclusive to animal owners, may be better discerned, and addressed and/or improved through different mitigation models to meet the needs of this and other groups. Viewing the challenges through the lens of the complex social microclimate, as described below in Fig. 2, affords such a perspective.

It is expected that detailed analysis following data collection will address these and other challenges actively identified in the data. The rationale for selecting PMT, and assessment of its ‘fitness’ as a framework to help achieve these goals is outlined below with a review of PMT over the last two decades.

### People and animal well-being

At one level, animal management in and around emergencies may appear to be an issue of animal welfare alone. But, as highlighted earlier, it is about people - as animals influence people’s decision making, and their fate, if adverse, adds to the burden of loss and the trajectories of recovery.

Current animal-owning household preparedness initiatives (by agencies such as the RSPCA) only target animal welfare outcomes, without articulating any possible subsequent benefits associated with human health and safety. Likewise, most of the literature about animal emergency management is about pets, and does not address the spectrum of animal ownership which exists in other sectors, such as farming, agribusiness, boarding and agistment (where animals are kept in the care of someone other than their owner, usually for a fee or reward) and other animal oriented SME’s. Discussion of non-companion animal loss is beginning to shift from an exclusive focus on financial or economic implications, with more consideration being given to psychological and emotional trauma. The more open discussion of mental health issues in the public realm generally, and a better understanding of the anguish and stress sustained by bushfire survivors in particular, has prompted greater consideration for farmers who manage and treat their burned or injured animals, or shoot and perhaps mass-bury their livestock, often after investing decades of skill and experience in genetic selection [18, 41, 85]. The farming community as an animal-owning group, widely recognised as resourceful and self-reliant, and highly experienced in animal husbandry and land management, could contribute significantly to assist other owner groups with less experience, and fewer skills.

### Future research – addressing the gap

None of the academic papers discussed above identify or document the animal owner/emergency responder interface as a resource to which PMT could be applied to improve self-efficacy or community efficacy. Nor do any scrutinise the potential to discover an untapped channel to improve hazard preparedness, or link possible broader societal gain with the potential contribution of facilitating animal owners and emergency responders working constructively together. In the context of bushfire, finding timely ways to help navigate a course for people *and* their animals to safety, could contribute to the saving of human life, and help avoid or reduce stress and mental ill-health which often occur following natural hazard emergencies [41, 63, 86]

The translation of knowledge into effective action - thereby lessening the impact of bushfire - is a fundamental necessity to create a culture of positive outcome expectancy and encourage confidence in bespoke bushfire survival plans – whatever their goal. Practical response over many years to awareness campaigns is widely acknowledged to be poor [9, 10, 12, 34, 77, 87, 88]. Figures reported by the South Australian Country Fire Service in their Annual Reports do indicate improvement, but numbers clearly demonstrate the persistently low correlation between awareness and positive behaviour change. In the 2014-15 Annual Report, 97% of the community responded that they understood the need for a plan, but only 41% (up from 25% the previous year) of respondents had actually taken the next step and created a plan suitable for their social microclimate [11]. Despite well-resourced bushfire prevention and survival campaign initiatives, progress in achieving behaviour change remains slow. The vision of this study is to endeavour to create a foundation of a *preceding* culture of preparedness as routine ‘business as usual’ – as routine as buying groceries or putting fuel in a motor vehicle. Ways to do this are the subject of later data analysis, and broadly involve examination of (i) flexibility of the workplace (ii) municipal fees and charges, and (iii) crop management among farmers.

Future research needs to address gaps in public policy and private practice to help people live and interact more safely in bushfire at-risk areas - often chosen for their natural beauty and nurturing surroundings; this includes routinely establishing emergency plans as relevant to the social microclimate, and, knowing when to leave. Although the best plans can fail – in itself a cause for psychological distress - the consequence of *not* planning could at worst lead to loss of human life, or long or short term morbidity. For people who experience a large scale bushfire, life will never be the same, regardless of personal impact. The social, environmental and economic costs post event can be immense. Animal owners and emergency responders are two groups well placed to contribute to research to help people live and interact more safely in bushfire at-risk areas.

### Towards a new expansion: Protection Motivation Theory – the last two decades

#### Other-directed applications in the health sector

In the last two decades, PMT has expanded beyond the realm of self-protection into vicarious other-directed health sector contexts such as the parent-child unit. In these studies, the use of PMT helped to understand parents’ behaviour, and enhanced health communications and messaging [89–91].

#### Expanding PMT into the environmental domain and natural hazards

PMT has been extended beyond the health sector into the environmental domain of climate change and slow-onset risk such as drought [92, 93]. Significantly, in these studies it was found to be useful in predicting adaptive behaviours across all aspects of the theory [94]. In a natural hazards context, PMT was used by Mulilis and Lippa [33] in a study of a highly realistic scenario (earthquake); they concluded that further research would help define PMT’s application.

Grothmann and Reusswig [34] expanded PMT in a quantitative study to describe the threat and coping appraisals in greater detail than Rogers’ original model, specifically pertaining to flood damage prevention. Included in their adaptation of PMT was recognition of *previous experience* (of flood), the reliability of known public protective infrastructure, the costs of private measures and maladaptive responses such as wishful thinking. Their findings concur with Tanner [39] that threat alone is not motivational, and that coping appraisal must be added in order to instill positive outcome expectancy and build response- and self-efficacy. Like Rogers, Grothmann and Reusswig believe PMT to have scope beyond its original application, and observe that a largely untapped advantage of using PMT with respect to natural hazards lies in its ability to better explain and understand human behavior. They note future research should target how to redress the current mismatch between public warnings and communication, and the uptake of appropriate preparedness and response behavior by private citizens.

#### Expanding other-directed PMT in natural hazards: issues of trust, complexity and response behaviour

Can PMT be applied to communities, groups, families or other collectives specifically including those with animals, exploring its application beyond the parent-child unit to variations of other-directed protective behaviour? As it evolves, dependable, robust, yet malleable social theory should be capable of contributing and responding to societal needs as they are identified. Increased understanding and implementation of more and different ways to narrow the bushfire awareness-preparedness gap will help reduce the human, economic and environmental toll of this natural hazard. Martin et al [95] observe that communities within high fire risk areas should not be viewed as “one homogenous” entity, but as comprising many different groups, each requiring particular information and assistance to successfully negotiate the threat of bushfire. Given this, PMT applied to the specific demographic of animal owners may help emergency responders anticipate how this group could behave within a scenario of threat and danger, and achieve a deeper mutual understanding and synergistic collaboration. Animal owners may learn how their own circumstances and bespoke solutions can help them reposition themselves to achieve a positive response- and self-efficacy.

After the 2009 bushfires in the state of Victoria, Australia, four theories, including PMT, were reviewed by Beatson [10]. The three others were Theory of Planned Behavior (TPB), Extended Parallel Processing Model (EPPM) [38] and Terror Management Health Model (TMHM). Each of these need, and deserve, further evaluation and research with respect to their contribution to public safety in bushfire natural hazards. However, as Beatson notes, both EPPM and TMHM could compromise preparedness actions and favour enhanced psychological resilience. TMHM also has a core focus on the influence of active, but non-conscious, thoughts of death on unsafe behaviours, which complicates further research. A limitation of TPB is that it does not differentiate between issues which may either facilitate or inhibit intention to engage in adaptive behaviour - which PMT does. Lindell and Perry’s revised model of Protective Action Decision Model (PADM) [96] appears to be potentially useful in the realm of risk communication. However, as the authors note, this theory needs further evaluation.

The current study utilised PMT because of its well-documented enduring adaptability and reliability. It is also relatively pragmatic and straightforward for lay people to understand and implement. Given the indisputable imperative of improving public preparedness and safety in bushfires, PMT offers the scope for new applications superimposed on an already well-tested and developed base.

**Fig. 1:**
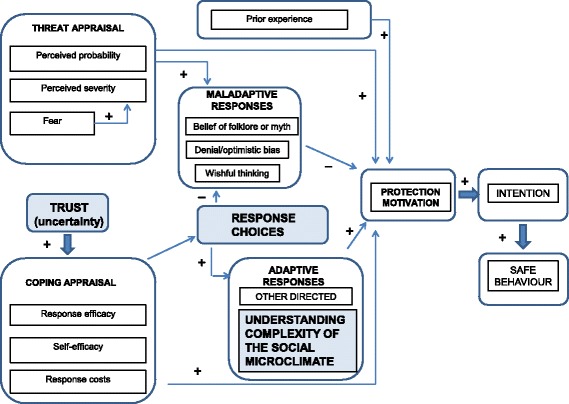
Expansion of Protection Motivation Theory. After Rogers [30] and adapted from Grothmann and Reusswig [34]. *Blue shading* indicates elements of the proposed PMT expansion. Permission to reproduce in an open access journal has been obtained from the copyright holder by the corresponding author

Beatson concluded by advocating the need to “stimulate targeted research which will lead to advances in community bushfire safety practice, and to find out which of the many constructs making up the theories are more important as determinants of bushfire-safety-enhancing behaviours”. This research responds to this need. It expands on Grothmann and Reusswig’s [34] PMT adaptation, adding the concepts of *trust and uncertainty*, *complexity of the social microclimate* and *response choices* (Fig. 1), to investigate its applicability to supporting and empowering animal owners and emergency responders in bushfire emergencies.

#### Trust and uncertainty

Paton [7, 74] describes *trust* as a critical element contributing to emergency preparedness. Examples include trust in agencies providing hazard information, trust in emergency services defending residents’ homes, and trust in oneself – the ability to respond appropriately in the face of danger. Community participation and organizational trust directly link to outcome expectancy, and these inter-relate as predictors of preparedness [12]. Trust, alongside accurate and timely information, can overcome uncertainty and avert the danger of maladaptive response [7]. Trust can, therefore, be assigned a place in the “coping appraisal” half of the PMT equation.

*Uncertainty* tends to bring community members together to find collective ways to cope, mitigate and survive hazards [12, 55]. Bockarjova and Steg [92] found that PMT contributed to understanding what motivates behavior in the “context of uncertainty”. As uncertainty increases, so too does the need to reliably trust sources of information. Community regard for emergency service providers may be defined by the amount of trust they have in that agency [12], and that culture of trust is influenced by past experiences with those agencies [12, 16, 88]. A high degree of organisational trust is more likely to increase self-responsibility for actions taken, and less likely to encourage negative outcome expectancy, preparedness inertia, and fatalistic or other unsafe behavior [12, 39, 51].

#### Trust pertaining to animal owners

Animal owners as a demographic comprise many subgroups. Owners of livestock, horses, companion pets, wildlife and animal related businesses are major categories. All animal owners need to trust emergency services and information providers that their animals, precious for whatever reasons, will be included and not excluded from emergency discussions – before, during and after the event. Owners also need to trust that responders will understand the importance of animals *to their owners*, regardless of the reason, and that separation, loss or injury of and to them will be traumatic at some level. Trust *can* be misplaced, which is why concurrent accurate information and knowledge sharing are needed. Usually trusted sources, such as a family member or experienced neighbour, may be themselves too traumatised, or be insufficiently knowledgeable about the presenting conditions to offer the guidance needed. Any subsequently compromised animal welfare may compound distress of the owner [47].

#### Complexity of the social microclimate

The heterogeneity of any given community or demographic as observed by Martin et al [95] and Gordon [51, 55] means that the social microclimate of a population often defines the degree of complexity inherent in any given context or collective, including that of animal owners. Among animal owners, this complexity will be influenced by the number, skill set and roles of individual family or work group members; the numbers and types of animals present, the underlying events of daily routine, and the presence, or otherwise, of a written, practised and understood bushfire survival plan. External influences could be relationships with neighbours, colleagues, and emergency services or other service providers, and all these will cause effective hazard preparedness and mitigation behaviour to vary. Complications can include simple logistics – the numbers of animals with respect to transport options and the time needed to evacuate or relocate animals to a place which may or may not have been pre-arranged.

When disaster is imminent, the usual differentiation among a community is temporarily lost and “debonding” – the loss of social fabric - is followed by a “fusion” into a homogeneous entity. This state is as much a threat as being de-bonded - and can preference maladaptive response [51]. Hence, concurrent social fusion may be superimposed on the social microclimate, and mask the real need for diverse coping appraisal for groups such as animal owners. Development of warnings, mitigation and response messaging protocols faces the challenge of achieving a balance between broad spectrum, generally applicable information, and providing enough bespoke material to reassure people that their individual circumstances are acknowledged and understood.

#### Complexity of family and household groups

A family’s preparedness and evacuation options are inversely proportional to the degree of complexity of their situation, but proportional to the time required to enact their plan (Pedler, T. & Prelgauskas, E. unpublished observation 2015). Where this includes the presence of animals, and recognising the need for bespoke mitigation options aligned with the social microclimate, broad sub-groups pertaining to animal ownership could include:

- the individual
- individual + household members (e.g. family, partner, children, dependent adults, elderly/disabled)
- individual + household members + animals
- individuals or community groups with attachment to non-owned animals at large, such as valued local wildlife
- self-activating or untrained volunteers

**Fig. 2:**
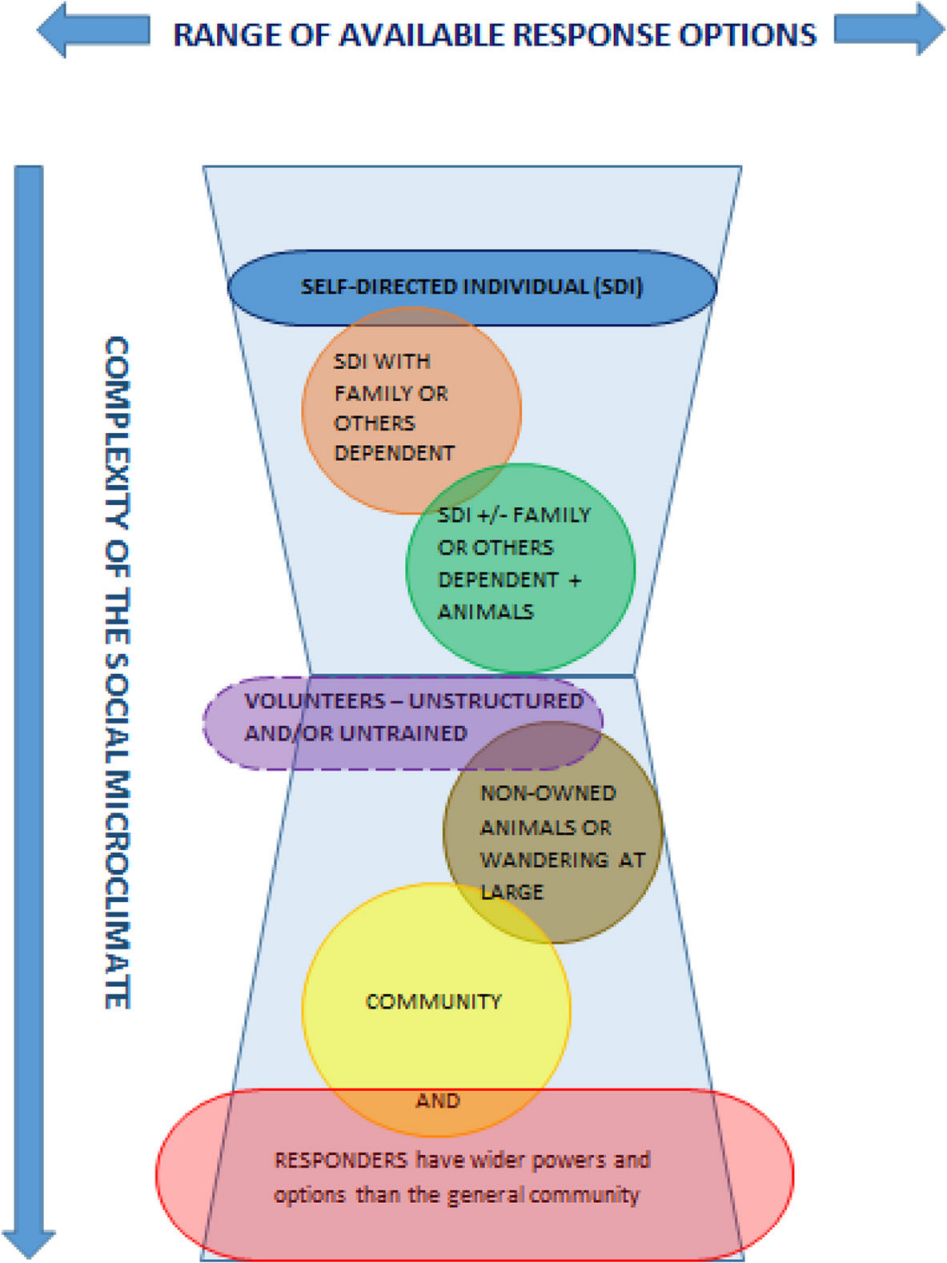
As conditions become more complex, response behavior options narrow, and are themselves more complex. Legend: Responders (*red*) have the widest range of powers and options available. The “Community” (*yellow*) has fewer but still quite extensive available options, and some of these will overlap with responder services such as Local Government, or Service clubs (e.g. Rotary, Lions). Community overlaps with non-owned animals because of resources potentially deployed in management, or because particular animals are highly valued. Untrained volunteers (*purple*) need management. People with animals (*green*) are a very diverse group with more complex needs than groups without animals (*orange*). An individual (*blue*) with no dependents or special needs should have the least complex microclimate, but does not have the broad legislated capability of emergency responders

The resulting other-directed actions can be included as part of an expanded PMT coping appraisal (Fig. 2).

**Individuals** – when managing only oneself in an emergency, current warnings, comprising comprehensive information from multiple sources should be sufficient for a physically and psychologically healthy adult to respond safely. While individual reactions will vary, most people support and help each other, and strive to maintain common values [55].

**Individuals + household members** – year-round outreach by fire services’ Community Engagement staff and public campaigns aims to help people understand that time needed for effective preparedness is a function of their personal and logistical resources, encouraging families to be proactive and engage in preparedness activities. This helps them recognise that effective mitigation measures *are* available, *and* can help them assess their own self-efficacy. More and better information leads to improved decision making [97] and helps avoid “highly aroused, emotionally motivated behavior” [55].

**Individual + household members + animals** – In this category, generalised directives may be insufficiently detailed, and bespoke solutions could be needed. This category is very broad. A family with animals such as a child’s pet(s), e.g. rabbits or guinea pigs, is very different from a parent, child and assistance dog, or family with children’s ponies or other “pet” livestock, a family business with animals such as boarding kennels, or a family of primary producers.

This category includes consideration of logistical problems such as multiple trips to transport animals, unsafe decisions leaving one person to move or manage stock, with or without adequate means of communication, and leaving too late – waiting “until we smell the smoke”. Dangerous consequences could include being caught in a fire front, motor vehicle accidents, injury and death (Prelgauskas, E. unpublished observation 2015).

**Individual or community with attachment to non-owned animals**, i.e. wildlife or animals wandering at large, which have two main effects. Firstly, populations of local wildlife may be particularly valued, and their survival or otherwise can buoy or depress a community, even in the presence of widespread property damage. Secondly, animals wandering at large could be present, and pose a risk, because they are local wild or feral animals, they have escaped because fencing infrastructure is destroyed, or because they have been intentionally released.

The difficulty of these situations, apart from the danger, lies in the fact that there is often very little that can be done in the short term, and this can be distressing. Wild animals, feral or endemic, require management with particular skills and resources which may necessarily take time to arrive. Loose animals may not be contained for days or weeks, and some may never be found. Injured animals may not receive immediate attention due to higher priorities. Owners may search for animals in vain, may discover them deceased or may be distracted, by their focused concern for animals, from taking the first steps towards their own recovery. The best, or perhaps only, option available may be to record the location of an animal loose or injured as accurately as possible, perhaps with a smartphone GPS or using local nomenclature. The very act of passing that information on to emergency services personnel can bring psychological comfort and peace of mind, and is also very useful for responders.

### Responders

While arguably not part of the social microclimate, the presence of responders defines the *milieu interiéur* of a natural hazard environment. In their interactions with animals, with or without their owners, responders will need to know how to manage these incidents, and what protocols exist to deal with them. The distraction of dealing with animals as an additional duty for responders should not occur and reduces their attention to core business, i.e. firefighting to protect life, property and the environment. From an operational perspective, an animal management presence on a Staging area, would allow responders to have a direct visual cue to enable rapid and accurate appraisal of the available animal emergency response services. This could assist in building collaborative interactions between responders and animal owners, enhance adaptive response – and boost responder morale [98]. Emergency responders having no choice but to ignore injured animals they may encounter is frequently identified by them as a source of distress, and has been the reason for closed and specific psychological debriefing post event (Klinberg, D. unpublished observation, and Walsh, D. personal communication 2015) [53, 98].

#### Complexity due to external others

Volunteers who may be untrained or unstructured in the context of social microclimate will also need management, and therefore consume resources [17, 99]. While acknowledged here, this group is outside the scope of the current paper.

#### Response choices: behaviour and personal safety

Human behaviour with respect to animals is much more complex than simply the welfare of the animal. Attachment to the animal, its place or use within the family, it’s value to a primary producer with or without value adding, or its importance as a performance animal are possible influences.

The presence of animals, and human attachment to them whether owned or otherwise, can influence attempts to ‘save’ animals with disregard for personal safety. Frequently this unsafe behaviour occurs because owners have not realized a threat is imminent, have left activating their plan too late, or possibly have succumbed to optimistic bias and denial. Dangerously, this can lead to a delayed attempt to flee, sometimes with animals in motor vehicles or trailers. Alternatively, owners may be away from home or off-farm at the time, or need more time to move large numbers of animals to safety. Owners might feel guilty if they have not prepared adequately for their animals, and this, superimposed on attachment to them can cause poor decision making such as rash attempts to return to their location. The presence of other sentient beings in an emergency may also cause a change in the behaviour of associated human beings. Generalised options are no longer viable, and the inadequacy of standard protocols could lead to maladaptive responses, including denial, belief of rumours and myth, simplistic judgement and wishful thinking.

Consequences of actions such as these could initiate a cascade of negative or even catastrophic events, leading to an avoidable risk to the lives of emergency responders or others [100]. In Australia, 42% of emergency services personnel, responding to a survey by Taylor et al [101], identified “Occasional or recurring” animal issues and 14% reported “significant or frequent” issues. The most problematic interactions occurred during the initial response, and around the rescue or relocation of animals at this time [101]. For example, overwhelming emotions can cause a limited focus on rescuing family or animals, and subsequent unsafe behavior such as an attempt to drive through a fire front. Response efficacy would be best achieved by advising fire fighters on the ground of the location of concern, enabling deployment of resources (fire crews, water bombers) to protect life and assets.

## Strengths, challenges & limitations

PMT appears to have the potential to encourage animal owners to better understand and be rigorous in their bushfire preparedness, and to help emergency responders engage with owners to build a reciprocally beneficial and collaborative relationship. PMT has a very practical and applied history: it has useful depth without being overly complicated. Previous research suggests its relevance and flexibility favours an application where positive on-ground outcomes are sought and required.

PMT has been successfully used in other-directed and natural hazard contexts in the past, and its adaptability has been established over four decades. This positive ‘track record’ could help convince responders of its merit, however each context for potential application of the theory presents unique challenges. These may include the social microclimate as discussed above, historic and cultural considerations in community interventions, openness to new approaches and the unpredictability of human responses.

Several theories are potentially applicable to the focus of this research [10]. Others are Theory of Planned Behavior (TPB), Extended Parallel Processing Model (EPPM) [38], Terror Management Health Model (TMHM) and Protective Action Decision Model (PADM) [96]. Further research could explore these, and their possible contribution to improve community safety and well-being.

Community intervention has its own inherent challenges. For example, after the Victorian bushfires in southern Australia in February 2009, recovery agencies found problems such as a pre-existing suboptimal relationship between a community and fire authorities, the presence of seasonal temporary residents, variable levels of hazard awareness, and false beliefs about the need for preparedness, all being barriers to effective engagement [88]. Similar issues could be found among hazard preparedness and response processes.

Unfamiliarity with PMT is another limitation. Most front-line, operational responders to bushfires would not be familiar with the theory or its principles. They may even be cautious or sceptical about the use of academic social theory in the practical context of firefighting. Given this, some responder education may be necessary to illustrate the value of PMT. Even so, a willingness to engage with PMT training, as a new and unfamiliar approach, could itself be a challenge.

Attachment to animals is a further potential barrier to the effective application of PMT. Emotive attachment to animals could override adaptive response in some circumstances, and could affect responders as well. Overcoming maladaptive response to ultimately achieve safe behaviour means that adaptive response needs to become instinctual and reflex, and adopting that assertion routine.

## Conclusion

The proposed expansion of PMT, as discussed in this paper includes *trust*, *complexity of the social microclimate*, and *response choices* as additive, interactive and testable, elements relevant to the subject groups of animal owners (of all kinds of animals) and emergency responders. This expansion connects an other-directed and natural hazards application, within a demographic and a context not previously researched. Owning animals has been identified as contributing to complicated, delayed or failed human evacuation, and as a trigger for untimely attempts to return to homes and properties to remove pets and other animals in the face of danger. As a diverse group consisting of nearly two thirds of Australia’s population, animal owners may helpfully contribute to contemporary emergency management problem solving. In parallel with emergency responders, new and effective paths to safer communities may be found.

An expansion of PMT, and its implementation as a tool to help emergency responders understand and work positively with animal owners, as detailed above, seems plausible and worthy of further investigation. The theory’s depth, in combination with its pragmatism, suggests its potential to be accepted by responders and to effectively improve procedures and outcomes in what can be traumatic and tragic circumstances, often with long term adverse social, environmental and economic consequences.

The literature suggests that PMT is robust, versatile and is still in widespread use after four decades. Its enduring relevance is a key indicator of its usefulness and dynamic applicability, and its evolution since 1975 suggests a baseline theory with considerable scope. PMT has offered solutions in several different realms of enquiry since 1983, as predicted by Rogers. Recently identified as a theory likely to have more to contribute, an expansion into a new application as proposed in this paper may determine solutions which will help achieve safer processes and response behavior, and narrow the bushfire awareness-preparedness gap.

## Abbreviations

EPPM: Extended parallel processing model
GPS: Global positioning system
PADM: Protective action decision model
PETS Act: Pets evacuation transportation standards act
PMT: Protection motivation theory
PTSD: Post-traumatic stress disorder
RSPCA: Royal Society for the Prevention of Cruelty to Animals
SES: State emergency service
SME: Small to medium enterprise
TBP: Theory of planned behaviour
TMHM: Terror Management Health Model
USA: United States of America
